# Differential expression of long non-coding RNA Regulator of reprogramming and its molecular mechanisms in polycystic ovary syndrome

**DOI:** 10.1186/s13048-021-00829-6

**Published:** 2021-06-21

**Authors:** Zhihong Zhang, Min Sang, Siqin Liu, Jing Shao, Yunjiang Cai

**Affiliations:** 1 Department of Obstetrics and Gynecology, General Hospital of Daqing Oilfield, Daqing, 163000 Heilongjiang China; 2 Gynecology Clinic, The First Hospital of Harbin, No.151 Diduan Street, Heilongjiang 150010 Harbin, China; 3 Laboratory Department, General Hospital of Daqing Oilfield, Daqing, 163000 Heilongjiang China; 4 Department of Psychology, Harbin Medical Univercity (Daqing), Daqing, 163319 Heilongjiang China

**Keywords:** Polycystic ovary syndrome, MiR-206, Regulator of reprogramming, Vascular endothelial growth factor

## Abstract

**Background:**

Polycystic ovary syndrome (PCOS) is a common endocrine disease in women of reproductive age. Multiple studies have shown that long non-coding RNAs (lncRNA) and microRNAs (miRNA) play a role in PCOS. This study aimed to explore the role and molecular mechanism of lncRNA -Regulator of reprogramming (lncROR) in PCOS.

**Results:**

Expression level of lncROR in PCOS patients was up-regulated, while level of miR-206 was down-regulated in comparison with control group (*P* < 0.001). Logistics regression analysis showed that lncROR and miR-206 were independent predictors of PCOS. The ROC curve showed that lncROR had a high diagnostic value for PCOS with an AUC value of 0.893. Pearson correlation coefficient indicated that the expression level of miR-206 was negatively correlated with the level of lncROR. CCK-8 assay and apoptosis assay revealed that downregulation of lncROR up-regulated the expression of miR-206, thereby inhibiting cell proliferation and promoting cell apoptosis. However, silencing the expression of miR-206 reversed the above effects caused by down-regulation of lncROR expression. Luciferase reporter gene assay suggested that there was a target relationship between lncROR and miR-206. VEGF was proved to be the target gene of miR-206.

**Conclusions:**

Highly expressed lncROR indirectly up-regulated the expression of VEGF by down-regulating the expression of miR-206, thereby promoting the proliferation of KGN cells and inhibiting apoptosis, and further promoting the development of PCOS.

## Introduction

Polycystic ovary syndrome (PCOS) is the most common endocrine disease in women of childbearing age [1], which is a group of clinical syndromes characterized by reproductive disorders, endocrine abnormalities and metabolic disorders [2]. The prevalence of PCOS is about 6% ~ 10% [3]. The most typical clinical features of PCOS are abnormal ovulation, hyperandrogenemia and polycystic ovarian changes [4]. The pathogenesis of PCOS is still unclear, but some studies have been proved that genetic [5] and environmental factors [6] play a role in the occurrence and development of the disease. A survey showed that metabolic disorders, obesity and type 2 diabetes (T2D) are considered to be the most important long-term problems of PCOS [7]. Correct diagnosis and timely intervention had the ability to effectively control the progression of disease and overcome the metabolic abnormalities. Therefore, it is of great significance to investigate the mechanism of PCOS and explore a new diagnostic method for PCOS under the current situation.

Long non-coding RNAs (lncRNAs), a type of RNA molecule with a transcript length of more than 200 nucleotides, are not encode proteins [8]. LncRNAs are involved in the regulation of transcriptional silencing, transcriptional activation, chromosomal modification and nuclear transport and other important processes [9], and are closely related to the occurrence, development, prevention and treatment of human diseases [10]. LncRNA Regulator of reprogramming (ROR) is located at 18q21.31 of chromatin and is also known as lincRNA-ST8SIA3 or ROR [11]. Studies have shown that ROR acted as a miRNA sponge to regulate the function of Oct4, Nanog and Sox2 in the self-renewal of human embryonic stem cells [12]. Shen et al. revealed that enhanced ROR-expression played a promoting role in the development of ovarian cancer [13]. In addition, Xu et al. found that the expression of ROR in the endometrium of patients with adenomyosis was significantly higher than that in the normal endometrium, and they also found that high expression of ROR promoted the proliferation of endometrial epithelial cells, thus speculated that ROR may promote endometrial hyperplasia [14]. Studies have shown that chronic anovulation in PCOS patients means long-term estrogen excess or progesterone deficiency, which is easy to cause atypical endometrial hyperplasia [15]. Therefore, we suspected that ROR was related to PCOS. However, the regulatory role of ROR in PCOS is still unknown. MiR-206 is located on chromosome 6 of skeletal muscle-specific Myomir, which is specifically expressed in muscles and considered to be an important regulator of muscle differentiation [16]. Marta et al. found that the expressions of miR-451a, miR-652-3p, miR-106b-5p and miR-206 in serum of PCOS patients were significantly down-regulated [17]. Vascular endothelial growth factor (VEGF) is an important regulator of angiogenesis, which promotes angiogenesis and endothelial cell division. VEGF expression was increased in tissues with active angiogenesis. Wang et al. showed a tendency of VEGF overexpression in serum of PCOS patients and patients with ovarian hyperstimulation syndrome (OHSS) [18]. All the above evidence suggested that ROR, miR-206 and VEGF may be correlated with each other and may play an important role in the occurrence of PCOS.

Therefore, in the present study, we investigated the expression levels of ROR and miR-206 in serum of PCOS patients and evaluated the correlation between ROR and miR-206. We preliminarily evaluated the regulatory effects of ROR, miR-206 and VEGF on PCOS and their regulatory mechanisms through in vitro cell experiments.

## Methods and materials

### Study population and serum collection

The subjects recruited in this study were women who came to The First Hospital of Harbin for reproductive problems and were diagnosed with PCOS. The diagnosis of PCOS meets at least two of the diagnostic criteria published by the European Society of Human Reproduction and Embryology in Rotterdam/American Society for Reproductive Medicine [19]. Diagnostic criteria were as follows: 1) Slight ovulation or anovulation; 2) Clinical manifestations of hyperandrogenism and hyperandrogenemia, total serum testosterone concentration > 0.5 ng/mL; 3) Ultrasonography indicated that there were 12 or more than 12 follicles with diameters of 2–10 mm on both or one ovary. Women in control group were healthy subjects with normal menstrual cycles and no clinical symptoms of PCOS. Age, body mass index (BMI) and hormone levels of all subjects were measured and recorded. Serum hormone levels were quantitatively determined by chemiluminescent immunoassay using UniCel DxI 800 automatic immune analyzer (Beckman Coulter, USA). The supernatant was centrifuged immediately after the venous blood was taken from the subjects and placed in a -80 °C refrigerator for later use.

This research program follows the ethical principles of human research in the Declaration of Helsinki and has been approved by the Ethics Committee of The First Hospital of Harbin. All recruited individuals in the study have signed informed consent.

### Cell culture

KGN cells were purchased from Shanghai Institute of Biochemistry and Cell Biology (SIBCB, Shanghai, China) and maintained in DMEM medium (Gibco, Carlsbad, CA, USA) containing 10% FBS (Gibco, USA) and 1% Penicillin/streptomycin (Gibco, USA). All cells were incubated in a humidified atmosphere with 5% CO_2_ at 37 °C condition.

### Cell transfection

KGN cells were seeded into 12 well plate at a density of 5 × 10^5^ cells/well and incubated overnight. Cells were transfected with negative control plasmids (oe-NC) or lncROR overexpression plasmids (oe-ROR), small interfering RNA negative control (si-NC) or small interfering RNA against ROR (si-ROR), miR-NC, miR-206 mimic or miR-206 inhibitor which were obtained from GenePharma (Shanghai, China) using Lipofectamine 2000 (Invitrogen, Carlsbad, CA, USA) for 48 h according to the manufacturer’s protocols.

### RNA extraction and qRT-PCR

Trizol reagent (Invitrogen, ThermoFisher Scientific, USA) was used for total RNA extraction. SuperScript II Reverse Transcriptase kit (Invitrogen, USA) and PrimeScript™ RT reagent Kit (Takara, Japan) were used to reverse transcribe the RNA of lncROR and miR-206 into cDNA, respectively. The expression levels of lncROR and miR-206 were determined using a miScript SYBR® Green PCR kit (Qiagen GmbH, Germany) in Applied Biosystems 7900 Real-Time PCR System (Applied Biosystems, Foster City, CA). The 2^−ΔΔCt^ method was used to calculate the relative expression level, and GAPDH and U6 were used as internal reference.

### Cell viability assay

Cell counting kit-8 (CCK-8) assay was used to assess the proliferation efficacy of KCN cells. Briefly, stably transfected KGN cells were seeded into 96-well plates at a certain density of 5 × 10^4^ cells/well and incubated at 37 °C overnight. At 0, 24, 48 and 72 h time point, CCK-8 solution was added to the cell culture plate and incubated in the dark for 4 h. The OD value at 450 nm was determined with a microplate analyzer. Each experiment was repeated in triplicate.

### Cell apoptosis assay

Flow cytometry analysis and Annexin V-FITC Apoptosis Detection Kit were performed to detect the cell apoptosis. After transfected for 48 h, KGN cells were harvested, were washed and resuspended. Annexin V-FITC solution (Invitrogen, USA) with a concentration of 0.25 μg/mL and PI solution (Invitrogen, USA) with a concentration of 1 μg/mL were added into cell suspension and incubate for 15 min in the dark. Finally, cell apoptosis was analyzed by a FACScan instrument (Becton Dickinson, Franklin Lakes, NJ, USA).

### Luciferase reporter gene assay

Studies have confirmed that lncRNAs acted as endogenous sponges of miRNAs in some diseases and play a role in regulating the disease process through specific binding or adsorption of miRNAs. The online program of StarBase v2.0 or Target-scan 7.0 predicted that lncROR and miR-206 or miR-206 and VEGF might have a linkage relationship or a target relationship. Luciferase reporter gene assay was used to verify this conjecture. The 3’-UTR fragments of lncROR or VEGF were respectively cloned into pGL3 luciferase reporter gene vectors to construct wild-type reporter vectors (ROR 3’-UTR-WT or VEGF 3’-UTR-WT) and mutated reporter vectors (ROR 3’-UTR-MUT or VEGF 3’-UTR-MUT). KGN cells (1 × 10^5^ cells/well) were seeded into 24 well plate. Subsequently, cells were co-transfected with the above vectors and miR-NC, miR-206 mimic or miR-206 inhibitor using lipofectamine 2000 (Invitrogen, Carlsbad, CA, USA) under the product instructions. After 48 h of co-transfection, cells were collected and luciferase activity in each group was determined by dual-luciferase reporting system (Promega, Madison, WI, USA). Rinilla luciferase as control gene.

### Statistical analysis

SPSS 21.0 software (SPSS Inc, Chicago, IL, USA) was used for statistical analysis. The difference in two groups was analyzed by Student *t*-test. One-way and two-way ANOVA followed by Tukey post hoc test were used for multi-groups comparison. Logistics regression analysis was applied to evaluate the role of different variables with PCOS. Pearson’s analysis was used for correlation analysis. A *P* less than 0.05 was indicative of a statistically significant difference. Data that conform to a normal distribution was expressed as a mean ± SD (standard deviation).

## Results

### Clinical characteristics of study population

The characteristics of PCOS patient group and control group were summarized in Table 1. A total of 68 patients with PCOS and 67 control subjects participated in this study. There was no significant difference in age, follicle-stimulating hormone (FSH), estradiol (E2) and prolactin (PRL) levels between the PCOS group and the control group. Notably, the BMI of the PCOS group was increased compared with the control group (*P* < 0.05), while the level of luteinizing hormone (LH) and testosterone (TES) were significantly increased in comparison with control group (*P* < 0.001).

**Table 1:** Clinical data of the study population

Variables	All subjects (*N* = 135)		*P* value
	Control (*n* = 67)	PCOS patients (*n* = 68)	
Age (years)	29.96 ± 4.49	30.35 ± 4.58	0.611
BMI (kg/m^2^)	21.93 ± 2.87	23.01 ± 3.11	0.037
FSH (mIU/ml)	6.74 ± 1.89	6.65 ± 2.06	0.797
LH (mIU/ml)	4.55 ± 2.09	9.91 ± 5.99	< 0.001
E2 (pg/ml)	46.71 ± 18.25	48.96 ± 16.38	0.452
PRL (ng/ml)	13.91 ± 4.91	13.71 ± 5.91	0.830
TES (ng/l)	0.422 ± 0.17	1.45 ± 0.38	< 0.001
AMH (ng/ml)	3.037 ± 1.05	10.89 ± 3.54	< 0.001

Note: *PCOS* polycystic ovary syndrome, *BMI* body mass index, *FSH* follicle-stimulating hormone, *LH* luteinizing hormone, *E2* estradiol, *TES* testosterone, *PRL* prolactin, *AMH* anti-Mullerian hormone. Data are expressed as n or mean ± standard deviation

### Expression level of serum lncROR was increased and miR-206 was decreased in PCOS patients

**Fig. 1:**
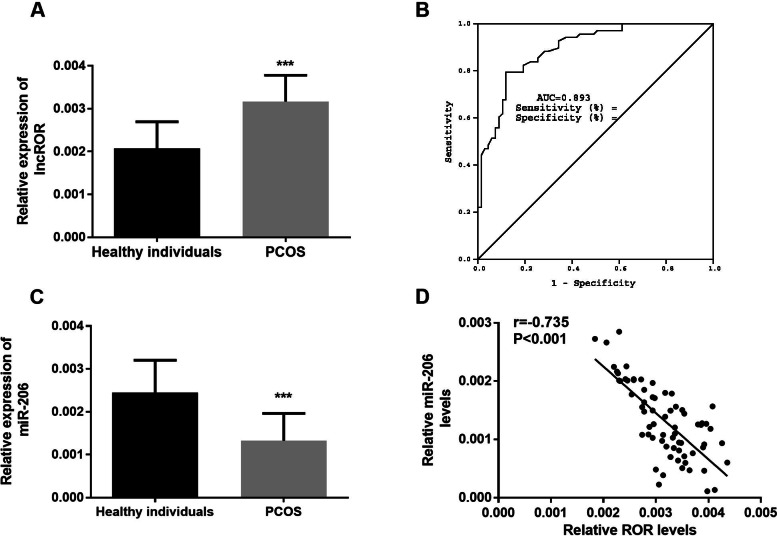
Clinical data analysis. **A** Relative expression level of lncROR in PCOS patient group was increased compared with control group (^***^P < 0.001, Student *t* test). **B** Relative expression level of miR-206 in PCOS patient group was decreased compared with control group (^***^P < 0.001, Student* t* test). **C** ROC curve analysis. LncROR has diagnostic value for PCOS, with high AUC value of 0.893. **D** Correlation analysis. The expression of miR-206 was negatively correlated with lncROR (*P* < 0.001, Student *t* test)

The expression levels of serum lncROR and miR-206 were determined by qRT-PCR. Compared with the control group, the level of lncROR was significantly increased in PCOS group (Fig. 1A, *P* < 0.001). However, the expression level of miR-206 in PCOS patients was significantly downregulated compared with control group (Fig. 1C, *P* < 0.001).

### ROC curve and correlation analysis

The diagnostic value of lncROR in PCOS was assessed by establishing an ROC curve. As shown in Fig. 1B, the curve had an AUC value of 0.893, with the sensitivity and specificity of 79.4% and 88.1%, respectively. These data indicated that lncROR had certain diagnostic value for PCOS. In addition, the correlation in lncROR level and miR-206 level as well as lncROR level and other related indicators were evaluated by Pearson correlation coefficient. In serum of PCOS patients, the relative miR-206 levels were negatively correlated with lncROR levels (Fig. 1D, *r* =—0.7351, *P* < 0.001). As shown in Table 2, the expression level of lncROR was positively correlated with BMI, LH and TES (*P* < 0.001).

**Table 2:** Correlation between lnc ROR and various indicators

Paraments	Correlation with lnc ROR (r)	*P-value*
Age (years)	0.235	0.054
BMI (kg/m^2^)	0.641	< 0.001
FSH (mIU/ml)	0.026	0.834
LH (mIU/ml)	0.716	< 0.001
E2 (pg/ml)	0.211	0.084
PRL (ng/ml)	0.072	0.560
TES (ng/l)	0.790	< 0.001
AMH (ng/ml)	0.618	< 0.001

*Abbreviations*: *BMI* body mass index, *FSH* follicle-stimulating hormone, *LH* luteinizing hormone, *E2* estradiol, *TES* testosterone, *PRL* prolactin, *AMH* anti-Mullerian hormone

### Logistics regression analysis

Logistics regression analysis was performed to determine the independent predictors of the occurrence of PCOS. Table 3 revealed that ROR (OR = 4.525, 95% CI = 1.151 – 17.799, *P* < 0.001) and miR-206 (OR = 0.043, 95% CI = 0.011 – 0.160, *P* < 0.001) were independently correlated with the occurrence of PCOS, respectively.

**Table 3:** Association of different variables with the occurrence of PCOS

Variables	OR	95% CI	*P* value
LncRNA ROR	4.525	1.151 -17.799	< 0.001
MiR-206	0.043	0.011 -0.160	< 0.001
Age (years)	0.941	0.324 – 2.737	0.912
BMI (kg/m^2^)	0.389	0.130 – 1.160	0.090
FSH (mIU/ml)	1.264	0.443 – 3.604	0.661
E2 (pg/ml)	1.403	0.486 – 4.054	0.532
PRL (ng/ml)	1.780	0.612 – 5.174	0.290

*Abbreviations*: *BMI* body mass index, *FSH* follicle-stimulating hormone, *E2* estradiol, *PRL* prolactin

### Knockdown of lncROR inhibits proliferation and promotes apoptosis of KGN

**Fig. 2:**
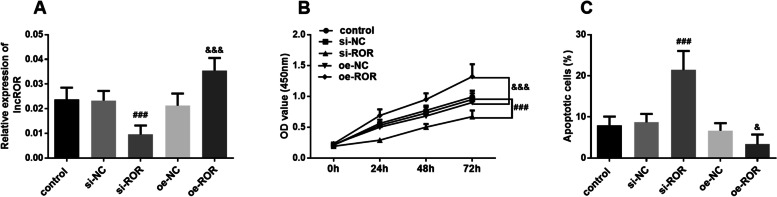
Effects of lncROR on the function of KGN cells. **A** Intracellular lncROR expression was regulated by in vitro cell transfected (^###^*P* < 0.001 vs. control group, ^&&&^*P* < 0.001 vs. oe-NC group, One-way ANOVA). **B** Cell proliferation of KCN cells was measured by CCK-8 assay (^###^*P* < 0.001 vs. control group, ^&&&^*P* < 0.001 vs. oe-NC group, One-way ANOVA). **C** Cell apoptosis of KGN cells was determined using flow cytometric analysis (^###^*P* < 0.001 vs. control group, ^&^*P* < 0.05 vs. oe-NC group, One-way ANOVA)

To explore the effects of lncROR on the proliferation and apoptosis of KGN cells in PCOS, cell transfection technology was used to regulate the expression of lncROR. The expression level of lncROR was significantly down-regulated after transfection with si-ROR, and likewise, the expression of lncROR was up-regulated after cells were transfected with oe-ROR (Fig. 2A, *P* < 0.001). CCK-8 assay showed that the cell viability of KGN cells which transfected with si-ROR was decreased compared with the control group (Fig. 2B, *P* < 0.001). In addition, flow cytometry results suggested that the number of apoptotic cells was significantly increased after KGN cells were transfected with si-ROR (Fig. 2C, *P* < 0.001). These results revealed that knockdown of lncROR suppressed cell proliferation and promoted cell apoptosis, and high expression level of lncROR is detrimental to PCOS.

### MiR-206 is a potential target gene of lncROR in KGN cells

**Fig. 3:**
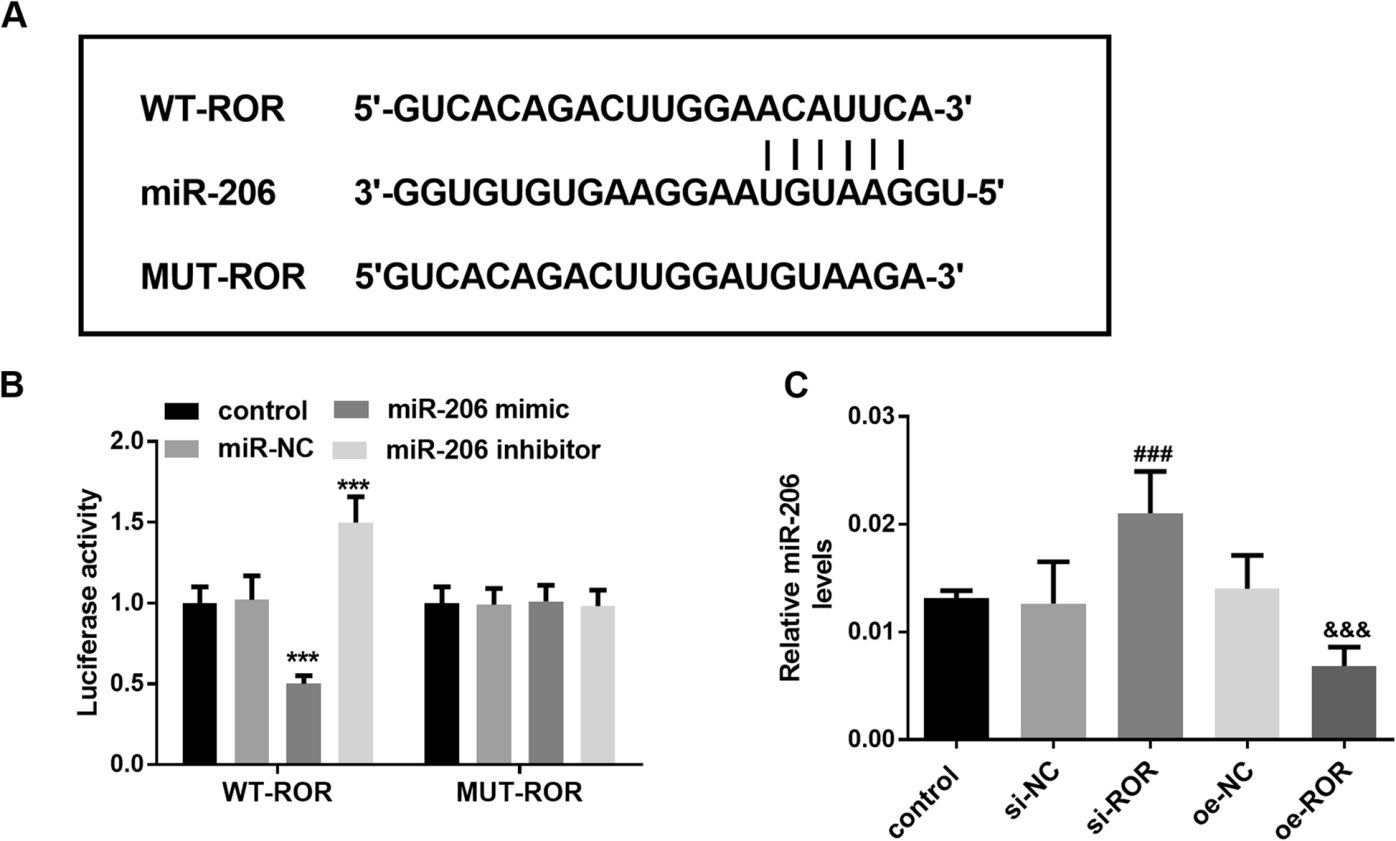
MiR-206 is a potential target gene of lncROR in KGN cells. **A** The complementary sequences of miR-206 and lncROR. **B** The interaction of miR-206 and lncROR was detected by luciferase reporter gene assay (^***^P < 0.01, One-way ANOVA). **C** Intracellular level of miR-206 in KGN cells (^###^*P* < 0.001 vs. control group, ^&&&^*P* < 0.001 vs. oe-NC group, One-way ANOVA)

To verify whether lncROR plays a regulatory role by inhibiting target miRNAs, we predicted the complementary sequence of lncROR and miR-206 through Starbase V2.0, and the results were shown in Fig. 3A. Luciferase reporter gene assay showed that luciferase activity was significantly decreased when cells were co-transfected with WT-ROR and miR-206 mimic, while luciferase activity was significantly increased after cells were transfected with miR-206 inhibitor (Fig. 3B, *P* < 0.001). Figure 3C showed that silencing of lncROR expression in KGN cells could up-regulate the expression of miR-206, while up-regulation of lncROR inhibited the expression of miR-206 (*P* < 0.001). These results jointly confirmed that lncROR could specifically bind to miR-206 in KGN cells and miR-206 may be a novel target of lncROR in KGN cells. Similarly, these results could also explain why miR-206 was lower in the serum of PCOS patients.

### Knockdown of miR-206 reversed the inhibition of proliferation and promotion of apoptosis induced by upregulation of miR-206 expression due to the silencing of lncROR

**Fig. 4:**
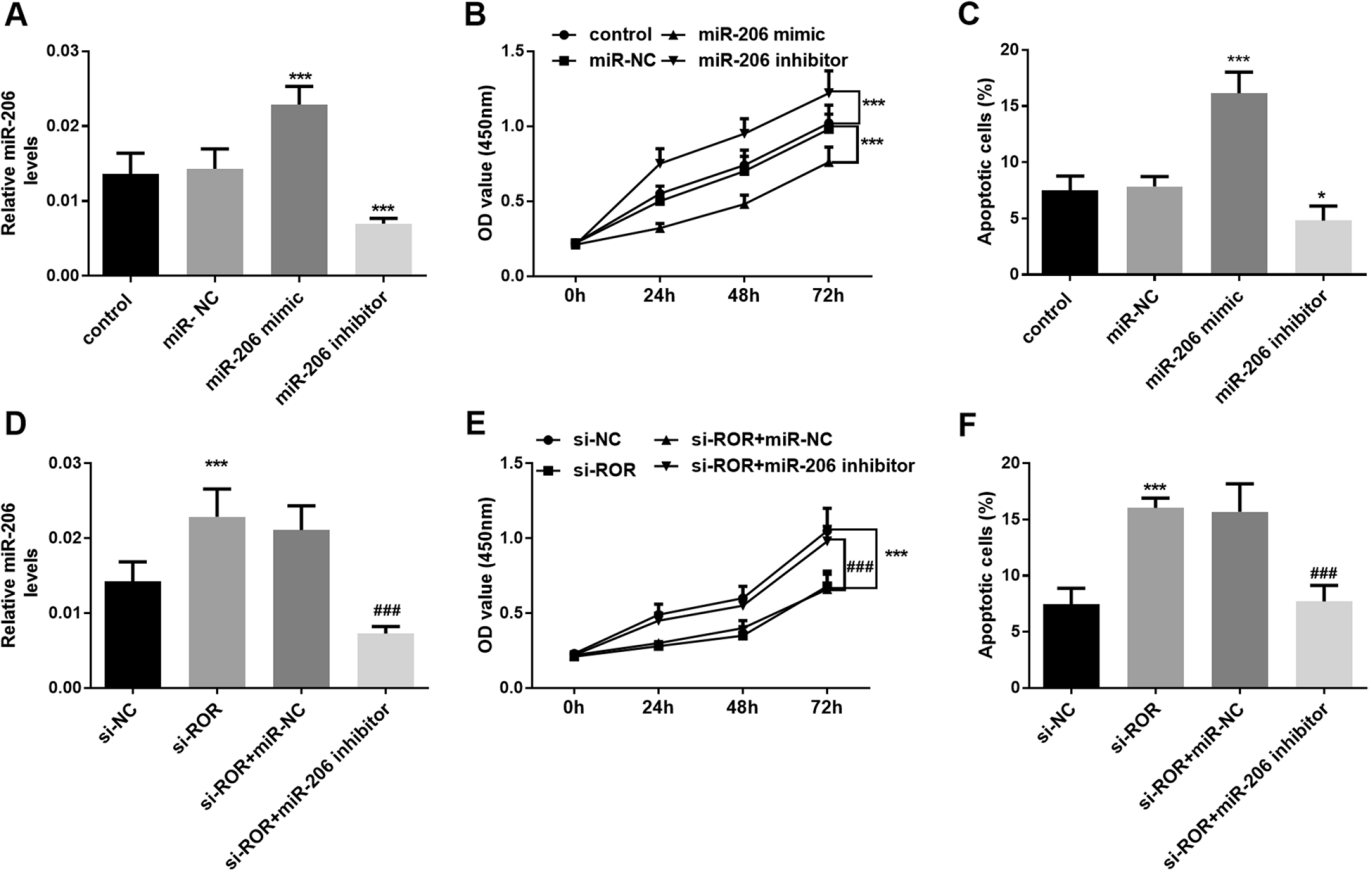
Effects of miR-206 on the function of KGN cells. **A** Intracellular miR-206 expression was regulated by in vitro cell transfected (^***^P < 0.001 vs. control group, One-way ANOVA). **B** Cell proliferation of KGN cells was measured by CCK-8 assay (^***^P < 0.001 vs. control group, One-way ANOVA). **C** Cell apoptosis of KGN cells was determined using flow cytometric analysis (^***^P < 0.001 vs. control group, One-way ANOVA). **D** Up-regulation of miR-206 could be achieved by silencing lncROR expression (^***^P < 0.001 vs. si-NC group, ^###^*P* < 0.001 vs. si-ROR + miR-206 inhibitor group, One-way ANOVA). **E** Cell proliferation of KCN cells was measured by CCK-8 assay (^***^P < 0.001 vs. si-NC group, ^###^*P* < 0.001 vs. si-ROR + miR-206 inhibitor group, One-way ANOVA). **F** Cell apoptosis of KGN cells was determined using flow cytometric analysis (^***^P < 0.001 vs. si-NC group, ^###^*P* < 0.001 vs. si-ROR + miR-206 inhibitor group, One-way ANOVA)

The expression of miR-206 in KGN cells was regulated by in vitro cell transfection and the effects of miR-206 on cell proliferation and apoptosis were investigated. The expression level of miR-206 was significantly up-regulated after cells were transfected with miR-206 mimic, while the level of miR-206 was significantly decreased after transfection with miR-206 inhibitor (Fig. 4A, *P* < 0.001). Up-regulation of miR-206 expression could significantly inhibit the proliferation and promote the apoptosis of KGN cells (Fig. 4B and C, *P* < 0.001). Additionally, it was worth mentioning that the expression level of miR-206 in KGN cells was significantly upregulated after transfection of si-ROR, while the above effects were reversed by miR-206 inhibitor after co-transfection of si-ROR and miR-206 inhibitor (Fig. 4D, *P* < 0.001). Both cell proliferation and apoptosis assay revealed the above results. Transfection of si-ROR could significantly inhibit proliferation and promote apoptosis, whereas these effects were significantly reversed by the miR-206 inhibitor. (Fig. 4E and F, *P* < 0.001). Together, the above results indicated that inhibition of lncROR expression in KGN cells led to overexpression of miR-206, thereby promoting apoptosis and inhibiting proliferation and controlling the progression of PCOS.

### VEGF is a target gene of miR-206 in KGN cells

**Fig. 5:**
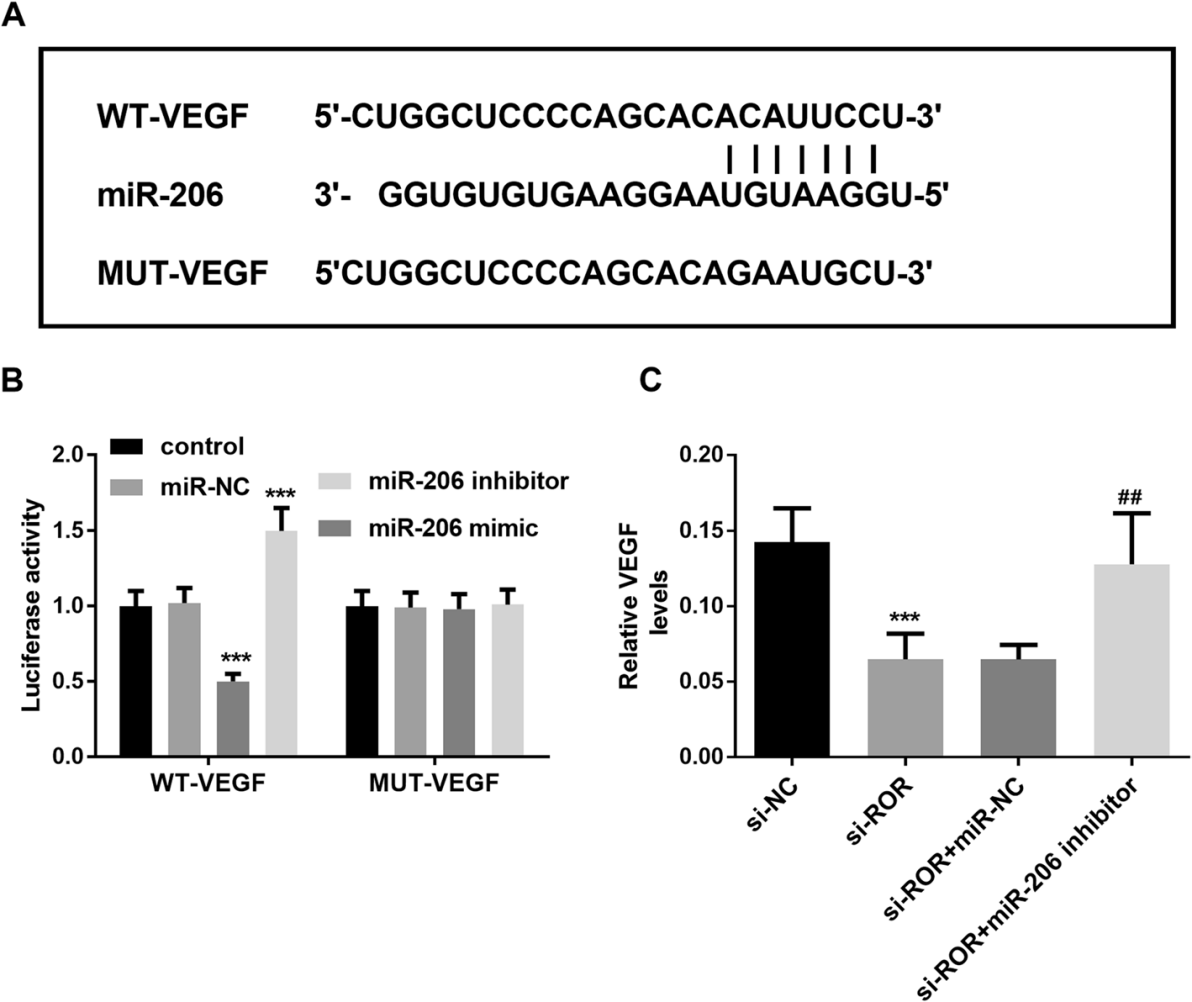
VEGF is a target gene of miR-206 in KGN cells. **A** The complementary sequences of miR-206 and VEGF. **B** The interaction of miR-206 and VEGF was detected by luciferase reporter gene assay (^***^P < 0.01, One-way ANOVA). **C** Intracellular level of VEGF in KGN cells (^***^P < 0.001 vs. si-NC group, ^##^*P* < 0.01 vs. si-ROR + miR-NC group, One-way ANOVA)

The online program of Target-scan 7.0 predicted that VEGF was the target gene of miR-206. Figure 5A showed the complementary sequence of miR-206 and VEGF. The luciferase reporter gene assay indicated that the luciferase activity in WT group was decreased after transfection with miR-206 mimic, and the luciferase activity was increased after transfection with miR-206 inhibitor, while this phenomenon was not observed in MUT group (Fig. 5B, *P* < 0.001). Figure 5C suggested that VEGF expression was down-regulated after transfection of si-ROR, while overexpression of VEGF appeared after co-transfection of si-ROR and miR-206 inhibitor (*P* < 0.001). The above results further confirmed the interaction in lncROR, miR-206 and VEGF, that is, miR-206 is a direct target of lncROR and VEGF. MiR-206 specifically binds to lncROR and was negatively regulated by lncROR, while the VEGF expression was negatively regulated by miR-206. In conclusion, lncROR regulated the expression of VEGF in KGN cells by sponging miR-206.

## Discussion

PCOS is a common endocrine disease, which affects women's ovulation in the short term and leads to fertility difficulties and other problems, while in the long term, it will seriously affect the health and quality of life of patients, such as obesity, abnormal blood lipid, abnormal metabolism, diabetes, and cardiovascular diseases. More and more studies are being carried out to combat this disease and the understanding of its pathogenesis could contribute to the diagnosis, prevention, and treatment of PCOS. In this study, the role of lncROR, miR-206 and VEGF in PCOS was explored through a series of experiments, and in vitro cell experiments confirmed that lncROR played a promoting role in the progression of PCOS by regulating the expression of VEGF through modulation of miR-206.

Our study found that compared with the control group, the expression level of lncROR in serum of PCOS patients was significantly increased, while the expression of miR-206 was significantly decreased. And the expression of miR-206 was found to be negatively correlated with lncROR level. This result is supported by the research of Marta et al. [17]. In the clinical data analysis table of all subjects, we found that the BMI of PCOS patients was significantly higher than that of the control group, and the hormone level expression of PCOS patients was abnormal, such as significantly increased in LH and TES. Moreover, the level of lncROR in PCOS patients showed significant positive correlation with the above abnormal indicators, including BMI, LH and TES. Besides, Logistics regression analysis showed that both lncROR and miR-206 were independent predictors of PCOS occurrence. Since KGN cells (Ovarian granulosa-like tumor cell line) has similar steroid activity to normal granulosa cells and expresses functional FSH receptors, many previous studies have used KGN cells as an in vitro evaluation model for PCOS [20]. Based on the above findings, we decided to use KGN cells to explore the possible molecular mechanisms of lncROR and miR-206 in PCOS. In the present study we found that down-regulation of lncROR could significantly inhibit the proliferation and promote apoptosis of KGN cells. A previous study revealed that lncROR was highly expressed in ovarian cancer tissues, and functionally induced epithelial mesenchymal transformation and promoted the proliferation and migration of ovarian cancer cells [21]. Additionally, luciferase reporter gene assay confirmed the sponging effect of lncROR on miR-206 and showed that the expression of lncROR was negatively regulated by miR-206. In recent years, lncRNAs have been considered as a type of ceRNA due to their interaction with miRNAs, and many studies have confirmed that lncRNAs play a role as ceRRNAs in the regulation of miRNAs. Through luciferase reporter gene assay, Li et al. confirmed that luciferase activity in WT-ROR group was significantly decreased after transfection of miR-145 mimic, suggesting that lncROR may be the ceRNA of sponge miR-145 [22].

PCOS is closely related to obesity [23] and insulin resistance [24], and most women with PCOS are obese or overweight, which in turn aggravates the degree of their endocrine disorders [25]. In a study of Alex et al., they found that the expression of miR-206 decreased in the blood circulation of obese subjects, while the expression increased in the blood of non-obese subjects [26]. Wu et al. reported that the injection of miR-206 into the liver of obese mice improved liver fibrosis and hyperglycemia [27]. All the above evidence confirmed that obesity was related to the reduction of miR-206 and the occurrence of PCOS. Considering the abnormal expression of miR-206 in PCOS patients, the effect of miR-206 on cells was also explored in this study. We found that knockdown of lncROR up-regulated the level of miR-206, which inhibited cell proliferation and promoted apoptosis, while these effects were reversed by miR-206 inhibitors. Dai et al. reported that miR-206 simulants inhibited the proliferation and migration of ovarian cancer cells in vitro and promoted apoptosis [28].

In addition, TargetScan 7.0 also predicted that miR-206 and VEGF had the binding site. Similarly, luciferase reporter gene confirmed that VEGF was the target gene of miR-206 and was negatively regulated by miR-206. The last result of this study showed that in KGN cells, silencing of lncROR down-regulated the expression of VEGF, while simultaneously knockout of lncROR and miR-206 could up-regulate the level of VEGF, which further indicated that the regulation of VEGF expression by lncROR was achieved by interacting with miR-206. In a new study of Shi et al., they confirmed that lncROR regulated tumor progression in renal cell carcinoma by regulating the miR-206/VEGF axis [29]. Their study helped us to better understand the interaction in lncROR, miR-206 and VEGF. Some studies have confirmed that VEGF could promote proliferation and migration of tumor cells and tissues and played an important role in tumor development [30]. In PCOS patients, VEGF concentration increased in ovarian tissues, and ultrasound Doppler blood flow monitoring showed that VEGF was significantly correlated with increased vascular density in tissues [31]. The above evidence confirmed that the high expression of VEGF has a negative effect on PCOS.

Overall, some limitations should be noted. First, we were unable to assess whether the expressions of lncROR and miR-206 in the follicles of PCOS patients and healthy people were consistent with the expressions in peripheral blood, there was no experimental data in this regard in our study. Second, PCOS is a lifelong disease of women, and the subjects included in this study belong to women of childbearing age. Therefore, it is unknown whether the results will be consistent with the current results if older and pre-menopausal women are enrolled. In view of the above problems, we believe that in the future scientific research work, in the early period of literature research, we should search more relevant literature. By referring to the previous research ideas, we can find the shortcomings of our own experimental design, and to design experiments more reasonably. In summary, it was confirmed that the expression of lncROR was increased while the expression of miR-206 was decreased in serum of PCOS patients compared with control women, and the expression levels of the two genes showed a negative correlation with each other. At the in vitro cellular level, we confirmed that inhibition of the expression of lncROR could up-regulate the expression of miR-206, and then down-regulate the expression of VEGF, thereby achieving the effect of proliferation inhibition and apoptosis promotion in KGN cells, and thus controlling the progression of PCOS. Although the current experiments had a certain significance in the analysis of the pathogenesis of PCOS, we still need to design more experiments or methods to further investigate the interaction in lncROR, miR-206 and VEGF.

## Data Availability

Corresponding authors could provide data and materials, if necessary.
